# Immunogenicity and protective efficacy of a native omp34 subunit vaccine against *Aeromonas hydrophila* in BALB/c mice: Identification of nitroblue tetrazolium as a correlate of protection within a One Health framework

**DOI:** 10.14202/vetworld.2025.4025-4045

**Published:** 2025-12-23

**Authors:** Rozi Rozi, Wiwiek Tyasningsih, Jola Rahmahani, Eduardus Bimo Aksono Herupradoto, Muchammad Yunus, Mohammad Anam Al Arif, Suryo Kuncorojakti, Annas Salleh, Suwarno Suwarno

**Affiliations:** 1 Veterinary Science Doctoral Study Program, Faculty of Veterinary Medicine, Universitas Airlangga, Surabaya, Indonesia; 2 Department of Basic Veterinary Medicine, Faculty of Veterinary Medicine, Universitas Airlangga, Surabaya, Indonesia; 3 Department of Veterinary Parasitology, Faculty of Veterinary Medicine, Universitas Airlangga, Surabaya, Indonesia; 4 Department of Farm, Faculty of Veterinary Medicine, Universitas Airlangga, Surabaya, Indonesia; 5 Department of Veterinary Anatomy, Faculty of Veterinary Medicine, Universitas Airlangga, Surabaya, Indonesia; 6 Department of Aquaculture, Faculty of Fisheries and Marine, Universitas Airlangga, Surabaya, Indonesia; 7 Department of Microbiology, Faculty of Veterinary Medicine, Universitas Airlangga, Surabaya, Indonesia; 8 Department of Veterinary Laboratory Diagnosis, Faculty of Veterinary Medicine, Universiti Putra Malaysia, Serdang, Malaysia

**Keywords:** *Aeromonas hydrophila*, nOmp34 subunit vaccine, IgG2a enzyme-linked immunosorbent assay, reactive oxygen species, phagocytic activity, protective efficacy, Life below water

## Abstract

**Background and Aim:**

*Aeromonas hydrophila* is a zoonotic, antimicrobial-resistant pathogen that causes significant losses in aquaculture and raises important One Health concerns. Outer membrane protein (OMP)–based subunit vaccines provide a targeted, antibiotic-sparing alternative to traditional bacterins, but validation across mammalian species remains limited. This study assessed the immunogenicity, safety, and protective effectiveness of a native ~34 kDa Omp34 (nOmp34) subunit vaccine in BALB/c mice, comparing it to a formalin-killed cell (FKC) vaccine, and examined immune factors that may predict survival.

**Materials and Methods:**

Female BALB/c mice (n = 13 per group) received subcutaneous injections of phosphate-buffered saline (PBS), FKC, FKC + incomplete Freund’s adjuvant (IFA), or native Omp34 + IFA on days 0, 14, and 28. Immune responses were assessed by measuring anti-Omp34 immunoglobulin (Ig)G2a levels via enzyme-linked immunosorbent assay, serum lysozyme activity, nitroblue tetrazolium (NBT) respiratory burst, and phagocytic activity at specified intervals up to day 42. On day 42, mice were challenged intraperitoneally with a lethal dose of *A. hydrophila*, causing 80% mortality, and observed for 14 days for survival, clinical scores, and body weight changes. Data analysis involved analysis of variance with Tukey *post hoc* tests, mixed-effects modeling, Spearman correlation, receiver operating characteristic curves, logistic regression, and Kaplan–Meier survival analysis.

**Results:**

By day 42, all immune biomarkers showed clear separation (nOmp34+IFA > FKC + IFA > PBS; p < 0.05). NBT demonstrated the strongest correlation with survival (ρ ≈ 0.90) and the highest predictive performance (Area under the curve [AUC] ≈ 0.80), exceeding IgG2a and phagocytosis (AUC ≈ 0.70). Post-challenge survival rates were 84.6% for nOmp34 + IFA, 61.5% for FKC + IFA, and 23.1% for PBS, corresponding to relative percent survival values of 80% and 50% compared to PBS. The direct comparison between nOmp34 and FKC revealed a favorable but not statistically significant survival benefit (p = 0.238). Vaccination was well-tolerated, with stable body weight, minimal reactogenicity, and no severe clinical events.

**Conclusion:**

The nOmp34 subunit vaccine elicited a strong, coordinated humoral and innate immune response, surpassing the matched bacterin in both efficacy and immune strength. NBT activity between days 35–42 proved to be a practical indicator of protection, aligning mechanistically with nicotinamide adenine dinucleotide phosphate -oxidase–mediated bacterial killing. These findings offer proof-of-concept for Omp34 as a scalable, antibiotic-sparing vaccine candidate and support its progression into aquaculture-relevant platforms within a One Health framework.

## INTRODUCTION

*Aeromonas hydrophila* is a widespread gram-negative opportunistic pathogen responsible for hemorrhagic septicemia in many aquatic species and occasional disease in mammals, thereby posing a significant zoonotic risk at the human–aquatic interface [1–4]. The increasing antimicrobial resistance among aeromonads further restricts treatment options and raises disease risks in aquaculture and public health sectors [5, 6]. In intensive freshwater production systems, *A. hydrophila* outbreaks lead to substantial economic losses, highlighting the urgent need for preventive measures rather than relying solely on treatments. This need is underscored by the approximately 65% rise in global antibiotic use from 2000 to 2015, which supports the case for vaccine-based, antibiotic-sparing disease control strategies [7]. Global stewardship tools, such as the World Health Organization (WHO) Access – Watch – Reserve (AWaRe) classification, promote this shift by encouraging the preferential use of Access antibiotics while limiting the use of Watch and Reserve categories [8, 9]. Adding to the problem, large proportions of prescribed antibiotics are excreted either unchanged or as active metabolites, maintaining selective pressure across interconnected aquatic and terrestrial ecosystems [10].

Subunit vaccines targeting outer membrane protein (OMP) offer an effective preventive alternative. These vaccines provide enhanced safety, precise antigenic targeting, and consistent manufacturing while efficiently activating antigen-presenting cells to stimulate T-helper–dependent B-cell responses [11]. Mechanistically, OMPs are conserved, surface-exposed β-barrel proteins whose solvent-accessible extracellular loops enable B-cell recognition and major histocompatibility complex-mediated antigen presentation [12–14]. Across numerous fish pathogens, OMP–based subunit vaccines have elicited strong immune responses and high protection levels, often matching or exceeding those of whole-cell bacterins. Multiple studies in teleosts consistently show robust relative percent survival (RPS)when specific OMPs are tested under controlled challenge conditions [15–17]. Simultaneously, advances in immunoproteomics and reverse vaccinology make it possible to systematically select conserved surface-exposed epitopes based on early manufacturability and cross-serotype effectiveness, thus speeding up translational development [18–20]. Several OMP antigens, including outer membrane protein W (OmpW), outer membrane protein A (OmpA), outer membrane protein N (OmpN), 46-kDa outer membrane protein (Omp46), outer membrane protein TS (OmpTS), and tolerance to colicins channel protein (TolC), have demonstrated broad immunogenicity and cross-protection in species such as blunt snout bream (*Megalobrama amblycephala*) [15, 16], red hybrid tilapia (*Oreochromis* sp.) [19], European eel (*Anguilla anguilla*) [21], common carp (*Cyprinus carpio*) [22], and channel catfish (*Ictalurus punctatus*) [23]. These findings highlight the translational potential of OMP–based subunit vaccines across diverse aquaculture species.

Among these candidates, Omp34 has emerged as a promising *A. hydrophila* antigen based on immunoproteomic screening [24]. However, its validation outside fish models remains limited, and a systematic test in a mammalian system is still needed to establish cross-phyla relevance [18, 25]. Furthermore, although OMP vaccines often correlate with improved survival, the relationship between humoral immunity (e.g., Immunoglobulin [Ig]G) responses and key innate mechanisms, such as serum lysozyme activity, reactive oxygen species (ROS)-mediated respiratory burst, and phagocytosis, has not been comprehensively studied in the context of OMP–based vaccination [15, 16, 26]. Filling these knowledge gaps is crucial for advancing One Health–oriented, antibiotic-sparing vaccination strategies for *A. hydrophila*.

Despite significant progress in developing OMP–based vaccines for *A. hydrophila*, most evidence comes from teleost models, with limited research in mammalian systems. Although several conserved OMPs (OmpW, OmpA, OmpN, Omp46, OmpTS, and TolC) show strong immunogenicity and high RPS in fish, the relevance of these antigens across different host species remains uncertain. Specifically, Omp34, repeatedly identified as an immunogenic and structurally conserved β-barrel protein in immunoproteomic screens, has not been systematically tested for its protective efficacy outside of fish, leaving its potential across phyla largely unexplored. Additionally, fundamental questions persist about how Omp34-driven humoral immunity (e.g., IgG2a) interacts with key innate immune mechanisms, including serum lysozyme activity, ROS production, and phagocytosis. These innate–humoral interactions are often associated with better survival in OMP–based vaccine studies, but they have not been fully characterized or connected to specific protection indicators in mammalian models. Furthermore, the existing *A. hydrophila* vaccine research rarely uses multivariable methods like ROC analysis or logistic regression to identify meaningful and practical protection markers. Overall, these gaps hinder the development of standardized, scalable, and antibiotic-sparing vaccine strategies that align with One Health priorities.

This study aimed to evaluate, for the first time, the immunogenicity, safety, and protective efficacy of a native ~34 kDa Omp34 (nOmp34) subunit vaccine in a mammalian model using BALB/c mice. Specifically, we sought to: (i) characterize the temporal development of Omp34-specific humoral immunity (IgG2a) and innate immune activation, including serum lysozyme, nitroblue tetrazolium (NBT) respiratory burst, and phagocytic activity; (ii) determine whether these immune responses lead to increased survival following a standardized lethal dose causing 80% mortality (LD_80_) *A. hydrophila* challenge; and (iii) identify practical correlates of protection through integrated statistical approaches, including correlation mapping, ROC analysis, and penalized logistic regression. By comparing nOmp34 with a formalin-killed bacterin under matched conditions, the study aims to establish the translational relevance of Omp34 as a scalable, antibiotic-sparing vaccine candidate and provide mechanistic insights that support One Health–aligned vaccine development for aquaculture and related public health interfaces.

## MATERIALS AND METHODS

### Ethical approval

All animal procedures were approved by the Institutional Animal Ethics Committee of Universitas Brawijaya (Approval No. 170-Kep-UB-2024), reported in accordance with Animal Research: Reporting of *In Vivo* Experiments (ARRIVE) 2.0 and applicable national regulations, and conducted at Universitas Airlangga’s Faculty of Veterinary Medicine under the same approved protocol. BALB/c mice were housed in individually ventilated cages at 22°C–24°C and 40%–60% relative humidity on a 12-h light/12-h dark cycle, with *ad libitum* access to AIN-93G chow (Surya Sains, Indonesia) and filtered water. Sterile corncob bedding and enrichment were provided according to facility standard operating procedures (SOPs). Welfare was monitored twice daily. At scheduled timepoints, body weight and a predefined 0–3 clinical score were recorded. Humane endpoints, including severe lethargy, refractory hypothermia, ≥20% weight loss, or clinical score ≥3, mandated immediate euthanasia using AVMA-aligned methods (CO_2_ inhalation followed by cervical dislocation), with confirmation of death.

### Bacterial strains and cultures

*A. hydrophila* AHS isolate was recovered from a diseased giant gourami (*Osphronemus goramy*) during outbreak investigations on aquaculture farms in Surabaya (East Java, Indonesia). Colonies were purified as single colonies and stored as a master seed (P0) at −80°C in 20% glycerol. All experiments used passages of ≤ P3. Cultures grew in TSB/TSA (Merck, Darmstadt, Germany); liquid cultures were incubated at 28°C, 180–200 rpm for 12–16 hours under aerobic conditions until reaching mid-log phase. Plates were incubated at 28 °C for 18–24 hours. Inocula were standardized by OD600 and verified by colony-forming units (CFU) back-titration. Phenotypic identification revealed gram-negative rods that were oxidase- and catalase-positive, with β-hemolysis on 5% sheep blood agar as a virulence indicator. Genotypic identification was carried out by 16S rRNA PCR (27F/1492R) followed by Sanger sequencing. For reporting, the isolate is designated as *A. hydrophila* sensu lato throughout this manuscript. No additional multilocus sequence analysis (MLSA), whole-genome sequencing (WGS), or average nucleotide identity (ANI) analyses were performed.

### FKC vaccine preparation

The master seed of *A. hydrophila* AHS (P0, −80°C, 20% glycerol) was revived in TSB (Merck, Darmstadt, Germany) and cultured to mid-log phase (OD_600_: 0.6–0.8) at 180–200 rpm and 28°C. Cells were harvested (5,000 × *g*, 10 min, 4°C), washed twice with phosphate-buffered saline (PBS; pH 7.4), resuspended to approximately 1 × 10^9^ CFU/mL, and inactivated with 0.4% (v/v) formalin for 24 h at 4°C with gentle mixing, followed by three washes in cold PBS. Sterility was confirmed by plating 100 μL on TSA (48 h at 28°C; duplicate plates monitored for up to 7 days); no colonies were observed. Vaccine strength was expressed as CFU-equivalents using an OD_600_–CFU calibration and verified by back-titration; each mouse received 1 × 10^8^ CFU-eq in 100 μL. Immediately before dosing, the aqueous FKC was emulsified 1:1 (v/v) with IFA using a two-syringe Luer-lock method (60–80 passes) to form a stable water-in-oil emulsion (drop test), kept on ice, and administered subcutaneously (100 μL, 26–27G). Any unused emulsion was discarded (no refreezing).

### Preparation of native Omp34 (nOmp34)

Cell pellets were resuspended in OM buffer (20 mM Tris–HCl, 2 mM EDTA, pH 7.5) and lysed on ice through probe sonication (30% amplitude; 10 s on, 20 s off; for a total of 4 min). Unbroken cells and debris were removed by centrifugation (5,000 × *g*, 10 min, 4°C). Total membranes were then pelleted by ultracentrifugation (100,000 × *g*, 60 min, 4°C). The pellet was washed once in 0.1 M sodium carbonate (pH 11.0) for 10 min at 4°C to remove peripheral proteins, re-pelleted under the same conditions, and resuspended in OM buffer. To enrich for outer membranes, suspensions were treated with 2% (w/v) N-lauroylsarcosine for 30 min at 20°C–25°Cwith gentle mixing. The sarcosyl-insoluble fraction was collected by ultracentrifugation (100,000 × *g*, 45 min, 4°C), washed twice with OM buffer or PBS (pH 7.4), and prepared for electrophoresis. Proteins were separated by sodium dodecyl sulfate–polyacrylamide gel electrophoresis (SDS–PAGE) using 12% polyacrylamide gels, with non-reducing Laemmli buffer. Gels were stained with Coomassie R-250; the band at 34–35 kDa (putative Omp34) was excised, electroeluted in Tris–glycine buffer at ~100 V for about 90 min, transferred immediately into PBS (pH 7.4) containing 0.02% (w/v) n-dodecyl-β-D-maltoside (DDM) for gentle refolding at 4°C. After buffer cleanup via dialysis or 10 kDa molecular weight cut-off (MWCO)spin-filtration, the protein concentration was measured by bicinchoninic acid (BCA) assay. The antigen was aliquoted, stored at −80 °C, thawed on ice before use, mixed gently, and not refrozen afterward.

### Band electroelution and refolding

The excised gel pieces were transferred to pre-soaked dialysis tubing and electroeluted in Tris–glycine buffer (25 mM Tris, 192 mM glycine; SDS-free) at 60–100 V for 60–90 min at 4°C. The eluates were concentrated (using a 10 kDa MWCO) and stepwise buffer-exchanged into PBS (pH 7.4) containing 0.02% (w/v) n-dodecyl-β-D-maltoside (DDM) with gentle, low-shear mixing to support detergent-assisted refolding. Protein concentration was measured by the BCA assay using bovine serum albumin (BSA) standards. Preparations were dispensed into single-use aliquots and stored at −80°C in PBS (pH 7.4) with 0.02% (w/v) DDM. For clarity, the antigen is called native/putative Omp34 (nOmp34). Just before use, aliquots were thawed on ice and mixed gently. Repeated freeze–thaw cycles were not allowed; any remaining volume was discarded. Lots were used within 14 days of preparation, with no drift in immunological readouts observed during this time. The residual ionic detergent was controlled within quality-control thresholds (MBAS <0.01% w/v; see Methods, Quality-control). No sonication or vigorous vortexing was performed after refolding. A future stability program (without expanding its current scope) will verify conformation via circular dichroism and native polyacrylamide gel electrophoresis (PAGE) and assess predefined criteria for freeze–thaw and extended storage at −80°C.

### Vaccine formulation and dosage

Each cohort received one of three formulations prepared under identical handling conditions: PBS vehicle control, *A. hydrophila* FKC expressed as CFU-equivalents, or the native Omp34 (nOmp34) subunit. Immediately before dosing, the aqueous antigen (nOmp34 in PBS, pH 7.4, 0.02% DDM; stored at −80°C in single-use aliquots) or the FKC suspension (held at 4°C on dosing days after sterility release) was emulsified 1:1 (v/v) with complete Freund’s adjuvant (CFA) for the initial dose and with incomplete Freund’s adjuvant (IFA; Sigma-Aldrich) for both boosts, using a two-syringe Luer-lock technique (60–80 low-shear passes) to create a stable water-in-oil emulsion (confirmed by drop test), kept on ice, and administered subcutaneously, 100 μL per mouse. The emulsions were prepared fresh and kept on ice; any unused material was discarded without refreezing. All injections were given subcutaneously over the dorsal scruff, using a 26–27 G needle under aseptic conditions; animals were monitored post-injection following welfare SOPs. The nOmp34 dose was 20 μg per mouse, chosen within the effective 10–50 μg range for OMP subunits in mice while conserving material; the FKC dose was 1 × 10^8^ CFU-equivalents in 100 μL, calculated from an OD_600_↔CFU/mL calibration and confirmed by back-titration. All groups followed a prime-boost-boost schedule on days 0, 14, and 28.

### Procedural quality-control

The purity of each antigen lot was confirmed by analytical SDS–PAGE with Coomassie staining and densitometry (acceptance ≥ 80%). Identity support for nOmp34 lots was provided by immunoblot using post-immune mouse sera (peptide-level identification (LC–MS/MS) was not performed in this dataset, and the antigen is reported as “putative nOmp34.”). Endotoxin was reduced by phase separation with Triton X-114 and buffer exchange into PBS (pH 7.4) containing 0.02% n-dodecyl-β-D-maltoside (DDM); the in vivo dosing lot met the kinetic Limulus amebocyte lysate acceptance criteria (< 1 EU μg^−1^). Serial dilution and concentration into DDM buffer were used to minimize SDS–PAGE carry-over; the dosing lot met MBAS acceptance criteria (<0.01% w/v). The final formulations contained ≤0.005% DDM per dose; enzyme-linked immunosorbent assay (ELISA) coatings and diluents were detergent-free.

### Western blot analysis

Purified native or putative Omp34 (nOmp34; 1–2 μg per lane) was separated using 12% SDS–PAGE and transferred to nitrocellulose via wet transfer (100 V, 60 min). Membranes were blocked with 3% BSA in TBS–0.1% Tween-20 (polysorbate 20; Sigma-Aldrich, St. Louis, MO, USA)for 1 h at 20°C–25°C, then incubated overnight at 4°C or for 1 h at 20°C–25°C with post-immune mouse sera (diluted 1:1,000–1:5,000 in TBS-T + 1% BSA), followed by three 5-min washes in TBS-T. They were then probed with HRP-conjugated anti-mouse IgG (diluted 1:10,000; 1 h at 20°C–25°C). After three additional 5-minute washes with TBS-T, bands were detected using enhanced chemiluminescence (ECL) and captured within the linear exposure range. Pre-immune sera served as negative controls. All gels and blots included molecular weight ladders (kDa) and clearly marked ~34–35 kDa bands; full, uncropped images and exposure details are provided in the figure legends. Identity assessments were based on size and immunoreactivity alone (see Quality-control); peptide-level identification via LC–MS/MS was not performed.

### Animals, sample size, and randomization

Thirty-nine female BALB/c mice (6–8 weeks old; 20–25 g; specific-pathogen-free [SPF]) were used; housing and welfare procedures are detailed under ethical approval. Sample size planning followed Federer’s criterion (r−1) (k−1) ≥ 15 for k = 3 groups, resulting in r ≥ 9 per group. To account for approximately 25% attrition and maintain power for survival analyses, 13 mice per group were enrolled (total n = 39). Animals were randomized to receive PBS, FKC + IFA, or nOmp34 + IFA using a computer-generated allocation sequence. Outcome assessors were blinded for immunological assays and post-challenge survival; clinical and injection-site scoring were also blinded according to predefined scales. Allocation concealment was achieved using coded vials and cage cards; assay plates used coded sample IDs with analysts masked until data lock. ELISAs were performed following predefined quality-control criteria (see ELISA methods). Immunizations were administered on days 0, 14, and 28 (prime, boost, and boost). Blood samples were collected via the lateral saphenous vein at pre-immune (day −1) and one day before each boost (days 13 and 27), with submandibular sampling permitted for confirmatory or terminal timepoints under institutional SOPs; volumes did not exceed 100 μL per time point, maintaining cumulative volumes within SOP-defined limits.

### Immunization schedule and blood sampling

Mice received 100-μL subcutaneous immunizations on day 0 (prime) and booster doses on days 14 and 28. Group A received PBS vehicle, Group B received formalin-killed *A. hydrophila* (1.0 × 108 CFU-equivalents per dose), and Group C received purified native OMP (~34 kDa; nOmp34, 20 μg per dose). To standardize adjuvant exposure, CFA was used for the prime, and IFA for both boosts. At each time point (D7, D14, D21, D35, and D42), a predefined subset (n = 3 per group) was bled (≤100 μL via submandibular or saphenous routes); cumulative volumes per animal complied with institutional limits. The D42 pre-challenge sample was provided to all mice, enabling linkage between baseline immune readouts (IgG2a, NBT, lysozyme, phagocytosis) and subsequent efficacy endpoints per animal.

### Indirect ELISA for anti-Omp34 IgG2a

Indirect ELISA for anti-Omp34 IgG2a was performed on high-binding plates coated with detergent-free nOmp34 (2 μg mL^−1^, carbonate–bicarbonate pH 9.6; 100 μL/well, overnight at 4°C), washed with PBS–Tween-20 (0.05%), and blocked with BSA (1–3%, 1 h, 37°C). Sera were diluted two-fold from 1:100 in PBS + 1% BSA and incubated for 1 h at 37°C (duplicates). Plates were washed 4–5×, then incubated with cross-adsorbed goat anti-mouse IgG2a–HRP (1:10,000) for 1 h at 37°C, followed by five washes before TMB development. TMB was developed (10 min to positive-control OD_450_: 0.7–1.0), stopped with 2 N H_2_SO_4_, and read at OD_450_ with an OD_650_ reference within 5 min. Each plate included blank, pooled pre-immune (negative), pooled post-immune (positive), and a fixed-dilution calibrator; acceptance required duplicate CV ≤ 15% and inter-assay calibrator CV <15%. Plate acceptance required blank OD <0.10, a positive-control titer within ±20% of its historical mean, duplicate CV ≤15% within the plates, and an inter-assay calibrator CV <15% across runs. Signals were baseline-corrected ΔOD = (OD_450_–OD_630_) sample − (OD_450_–OD_630_)_blank; For figures that report IgG2a in percent, “reactivity (%)” was defined as 100 × (ΔOD_sample / ΔOD_calibrator) at the fixed 1:100 dilution. In parallel, the endpoint titer was the reciprocal of the highest dilution with ΔOD ≥ (mean plate negative + 3 standard deviation [SD]) or, if the plate negative was unavailable, ΔOD ≥0.20 above blank; titers were log_10_-transformed and summarized as GMT ±95% confidence intervals (CI). Rationale for isotype selection: we prioritized IgG2a because, in mice, it can be consistent with Th1-skewed humoral responses and FcγRIV-mediated effector mechanisms, although confirmation of Th1 polarization would require additional isotypes or cytokines. IgG2a was evaluated together with NBT, phagocytosis, and lysozyme to align with opsonophagocytic mechanisms. ELISA wells were technical duplicates (CV ≤ 15%) and were averaged at the animal level (no pseudoreplication) prior to inference. For days 7 and 14, groups were compared by one-way analysis of variance (ANOVA) with Tukey’s Honestly Significant Difference (HSD) (α = 0.05); superscript letters denote Tukey groupings (same letter = ns, different letters = p < 0.05).

### Serum lysozyme activity

Turbidimetry was conducted using *Micrococcus lysodeikticus* (0.2 mg/mL; 0.05 M phosphate buffer, pH 6.2; 37°C). Serum (10 μL) was mixed with 250 μL substrate; OD^450^ decrease was measured every minute for 5 minutes. One unit was defined as a 0.001 decrease in OD_450_ per minute; results were referenced to a hen egg-white lysozyme standard curve (U/mL). Biological replicates consisted of individual mice (n = 3 per group at each time point: days 7, 14, 21, 35, and 42); technical replicates involved duplicate wells; sample volumes per draw were ≤100 μL and within institutional limits.

### Respiratory burst (NBT assay)

Leukocytes were prepared with ammonium–chloride–potassium lysis buffer, washed, counted, and seeded at 1 × 10^5^ cells per well in clear, phenol-red-free 96-well plates. The cells were incubated with nitroblue tetrazolium (NBT, 0.2 mg/mL) in the dark at 37°C for 30 min with or without 100 nM phorbol 12-myristate 13-acetate (PMA) as a stimulator. After incubation, cells were washed twice with PBS to remove unreacted dye, and formazan deposits were dissolved in dimethyl sulfoxide (DMSO) or a mixture of 2 M KOH in DMSO (1:1). Absorbance was measured at 620 nm using a microplate reader, with the blank-well background subtracted. Biological replicates consisted of individual mice (n=3 per group per time point: days 7, 14, 21, 35, and 42), and technical replicates were duplicate wells. Results were expressed as OD_620_ normalized to unstimulated controls, with a coefficient of variation (CV) ≤ 15%. Trypan blue exclusion confirmed cell viability > 90%. As specificity controls, superoxide dismutase (SOD; 300 U mL^−1^) or Tempol (4-hydroxy-TEMPO; 0.5 mM) were added 10 min before NBT incubation.

### Phagocytic activity

Peripheral leukocytes were isolated by lysing red blood cells from 50 μL of blood. The cells were then mixed with heat-killed, opsonized *A. hydrophila* (MOI 10–20:1) and incubated for 30 min at 28°C with gentle mixing. After washing, extracellular bacteria were either quenched with 0.2% trypan blue or removed through gentamicin protection (100 μg mL^−1^, 30 min). Cells were cytocentrifuged, fixed with methanol, and stained with Giemsa. A minimum of 200 monocytes or neutrophils were counted at 400× magnification by a blinded observer. Biological replicates consisted of individual mice (n = 3 per group per time point: days 7, 14, 21, 35, and 42), while technical replicates were duplicate smears.

Phagocytosis (%) = (total phagocytes ingesting bacteria) ÷ (total phagocytes) × 100.

### Safety and tolerance monitoring

Safety was prospectively monitored through body weight, blinded clinical scoring, and injection-site reactogenicity. Body weight: day 0 (pre-dose), days 1–7 after each immunization, then at scheduled intervals until challenge; deviations of ≥10% from baseline prompted veterinarian review, and deviations of ≥20% were considered a humane-endpoint per facility SOPs. Clinical status: Assessed on a blinded 0–3 scale; 0 indicates normal activity/grooming; 1 indicates mild piloerection or slightly reduced activity without dehydration; 2 indicates moderate lethargy, piloerection, and/or reduced intake; 3 indicates severe debility or moribund, assessed twice daily during the first 7 days post-dose and daily thereafter. Injection-site reactions: graded on a blinded 0–3 scale on days 1–7 (0 = none; 1 = mild erythema and/or ≤2 mm swelling; 2 = 2–5 mm swelling/induration; 3 = >5 mm swelling, ulceration, or necrosis); inspection was visual only. Animals meeting pre-specified humane-endpoint criteria (e.g., clinical score ≥2/3 or weight loss thresholds) were euthanized immediately using AVMA-approved methods (CO_2_ inhalation followed by cervical dislocation), with death confirmation. All monitoring was performed by blinded assessors under predefined humane-endpoint criteria to ensure an unbiased and ethical evaluation. Serum biochemistry and hematology were not included in this discovery-phase dataset but were incorporated into the predefined follow-up safety plan (ALT, AST, BUN/creatinine, CK ± basic hematology) within fixed post-dose and post-challenge windows. The 14-day post-challenge monitoring schedule (twice daily checks, blinded) is detailed in Section 10.3; the results summarize the safety outcomes. Necropsy observations were limited to gross inspection to support clinical adjudication.

### Challenge procedure and efficacy endpoints

Thirty-nine BALB/c mice (13 per group) were randomly assigned to the PBS control, FKC + IFA, or nOmp34 + IFA groups. Clinical scoring and endpoint adjudication were performed by a blinded team. The *A. hydrophila* AHS challenge stock was revived from the −80°C master seed (≤P3), grown in TSB to mid-log phase (OD600: 0.6–0.8; 180–220 rpm; 28°C), pelleted (4,000–6,000 × *g*, 10 min, 4°C), washed twice with PBS (pH 7.4), and resuspended on ice. Target inoculum density was determined using an OD600↔CFU/mL calibration and verified on the challenge day by back-titration on TSA (10-fold serial dilutions; triplicate plates; 18–24 h at 28°C). Challenges proceeded only if the back-titer was within ±10% of the target. TSA, tetrasodium sulfate, was used for the inocula, which were kept on ice and used within 2 hours; any remaining inocula were discarded.

At 48 h after the day 42 pre-challenge bleed, mice were challenged intraperitoneally with a near-LD80 dose (~1 × 107 CFU in 100 μL PBS). Animals were monitored at least twice daily for 14 days with blinded clinical scoring (0–3, predefined signs) and scheduled body weight recording. Prespecified humane endpoints (e.g., ≥20% weight loss, severe lethargy/ataxia, or clinical score ≥2/3) triggered immediate euthanasia using AVMA-aligned methods (CO_2_ inhalation followed by cervical dislocation), with death confirmation. Outcome assessors remained blinded to group allocation throughout monitoring and endpoint adjudication.

The primary efficacy endpoint was survival until day 14, as summarized by the Kaplan–Meier test. Group differences were evaluated with a two-sided, global log-rank test, followed (if applicable) by Holm-adjusted pairwise comparisons. Effect sizes were estimated using Cox proportional-hazards models (reference = PBS) with hazard ratios (HR) and 95% CI, and the proportional-hazards assumption was checked via Schoenfeld residuals. The survival rate (SR, %) on day 14 was calculated as follows:

SR (%) = (Number of surviving mice/Initial number of mice) × 100

Vaccine efficacy was assessed as RPS, which is defined as follows:

RPS (%) = [1 – (mortality in vaccinated group/mortality in control group)] × 100

Post-challenge organ burdens (log_10_ CFU/g), reisolation, and histopathology were not conducted in this study.

### Statistical analysis

Analyses were conducted using IBM SPSS Statistics 27 (IBM Corp., NY, USA). Data are presented as mean ± SD or median (IQR). Normality was evaluated with the Shapiro–Wilk test, and homogeneity was checked using the Levene test. A log_10_ transformation was applied when appropriate, such as for titers and OD_620_, to stabilize variance and improve normality. ELISA wells were technical duplicates with a coefficient of variation ≤15 percent and were averaged at the animal level before analysis.

Unless otherwise specified, longitudinal endpoints were analyzed using mixed-effects models that included fixed effects for group, time, and their interaction, along with a random intercept for each subject; random slopes were added if supported by diagnostics. Pairwise contrasts were conducted with Tukey adjustment on estimated marginal means. For day-specific summaries, Welch ANOVA was used when variances were unequal, or Kruskal–Wallis with Dunn tests when distributional assumptions were not met. Multiplicity was controlled with Tukey’s test for within-day pairwise comparisons and Holm’s test for families of hypotheses. Two-sided alpha was set at 0.05. Exact p-values were reported to three decimal places; values <0.001 were reported as p < 0.001.

Respiratory burst activity (OD_620_) was analyzed using a mixed-effects framework, with effect sizes summarized as omega squared (ω²) and 95% CI. Phagocytosis was examined in two parts: the phagocytic rate (% of phagocytes ingesting bacteria), modeled with a binomial generalized linear mixed-model with a logit link (switched to a beta-binomial when overdispersion occurred); and the phagocytic index (particles per cell), modeled with negative binomial or gamma mixed models with a log link, or with a linear mixed-model after log_10-_transformation when assumptions were satisfied. Lysozyme activity and other right-skewed serum measurements were log_10-_transformed and analyzed within the same mixed-effects framework. Survival data were summarized using the Kaplan–Meier method and compared with a global log-rank test. Effect sizes were estimated using Cox proportional-hazards models (reference = PBS), reporting HR and 95% CI, with proportional-hazards assumptions verified using Schoenfeld residuals.

Post hoc power was estimated descriptively using the Schoenfeld approximation for survival and the Hanley–McNeil variance for area under the curve (AUC) relative to 0.5. Missing-data handling followed a prespecified plan: observations that failed quality-control or were censored by humane endpoints were not imputed; all available data were analyzed using listwise deletion for model fits, and the effective n per endpoint and time point is indicated in the Results or figure captions.

## RESULTS

### Biochemical and phenotypic characterization of the AHS strain

The biochemical profile of the AHS strain aligns with a typical *Aeromonas* phenotype: gram-negative rods, oxidase- and catalase-positive, motile in semisolid agar, fermentative in O/F-glucose, nitrate-reducing, resistant to the vibriostatic agent O/129 (150 μg), growth at 0%–4% NaCl but not at 6%, and an A/A TSI reaction with gas and no H_2_S. Enzymatic tests (DNase, gelatinase, and aesculin) and β-hemolysis were positive. Carbohydrate utilization showed a broad fermentation pattern (glucose, sucrose, lactose, arabinose, galactose, mannitol, and maltose). Overall, the biochemical profile demonstrated 96% biochemical concordance with the Janda and Abbott [1] diagnostic scheme (Table 1).

**Table 1:** Phenotypic characterization of the AHS strain versus the Janda and Abbott [1] reference panel.

Test	Method/Medium	AHS results (This study)	Reference [1]	Match
Gram stain	Standard gram	Gram-negative rods	Gram-negative rods	Yes
Oxidase	Kovács	Positive	Positive	Yes
Catalase	3% H_2_O_2_	Positive	Positive	Yes
Motility	Semisolid (0.3%–0.4% agar)	Motile	Motile	Yes
O/F-glucose	Hugh–Leifson	Fermentative	Fermentative	Yes
Nitrate reduction	Standard nitrate	Positive	Positive	Yes
Vibriostatic agent O/129	Disk 150 μg on MHA	Resistant	Resistant (differentiates fromVibrio)	Yes
NaCl tolerance	0%–4% NaCl	Growth	Growth	Yes
High tolerance of NaCl	6% NaCl	No growth	No growth	Yes
Indole	Kovács	Positive	Positive (often in *A. hydrophila*)	Yes
Methyl red (MR)	MR-VP	Negative	Negative	Yes
Voges–Proskauer (VP)	MR-VP	Negative	Negative	Yes
Citrate	Simmons citrate	Positive	Often positive	Yes
TSI slant	Triple sugar iron	A/A with gas; H2S −	A/A ± gas; H2S usually −	Yes
DNase	DNase agar	Positive	Positive	Yes
Gelatinase	Gelatin liquefaction	Positive	Positive	Yes
Aesculin hydrolysis	Bile aesculin	Positive	Positive	Yes
Hemolysis	5% sheep blood agar	β-hemolysis	Often β-hemolysis	Yes
Growth at 37°C	TSA	Growth	Growth (common)	Yes
Growth at 42°C	TSA	No growth	No growth	Yes
Glucose	Red phenol broth (1%)	Positive	Positive	Yes
Arabinose	Red phenol broth (1%)	Positive	Variable	Variable
Galactose	Red phenol broth (1%)	Positive	Positive	Yes
Sucrose	Red phenol broth (1%)	Positive	Variable	Yes
Lactose	Red phenol broth (1%)	Positive	Variable	Yes
Mannitol	Red phenol broth (1%)	Positive	Positive	Yes
Maltose	Red phenol broth (1%)	Positive	Variable	Yes
The overall biochemical concordance		26	27	96%

### Molecular confirmation and taxonomic placement of the AHS strain

**Figure 1:**
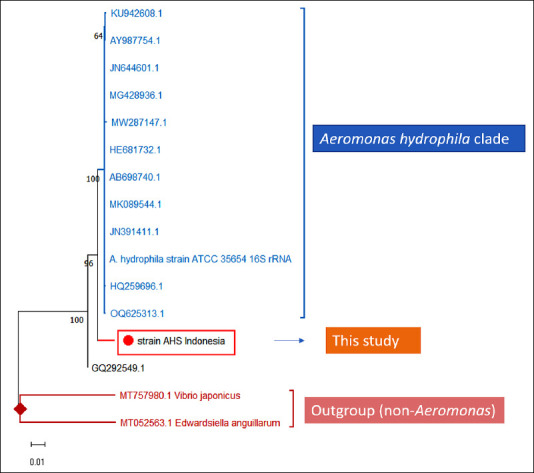
Neighbor-joining phylogeny based on 16S rRNA sequences (1,000 bootstrap replicates). The blue square indicates the isolate from this study (AHS, Indonesia). Black circles represent reference *Aeromonas* sequences, and red circles denote outgroup taxa (*Vibrio japonicus* MT757980.1; *Edwardsiella anguillarum* MT052563.1). The brackets highlight the Ae*romonas hydrophila* cluster. Bootstrap values below 70 are shown for completeness and should be interpreted cautiously. Scale bar: 0.01, substitutions per site. Reference GenBank accession numbers: KU942608.1, AY987754.1, HE681732.1, MG428936.1, AB698740.1, JN644601.1, MK089544.1, JN391411.1, HQ259696.1, OQ625313.1, MW287147.1, and GQ292549.1.

A neighbor-joining phylogeny of the *16S rRNA* gene (K2P, 1,000 bootstrap replicates), rooted with *Vibrio* and *Edwardsiella*, placed the AHS isolate within the *A. hydrophila sensu lato* group and in a cluster that includes ATCC 35654 (Figure 1). The AHS isolate shared 100% 16S identity with ATCC 35654, reinforcing this placement, while the supporting internal node carried modest bootstrap support (45), reflecting the known limited species-level resolution of 16S within *Aeromonas*.

### Identification of a ~34 kDa OMP candidate

**Figure 2:**
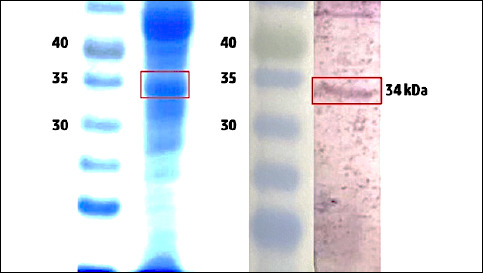
sodium dodecyl sulfate–polyacrylamide gel electrophoresis (SDS–PAGE) and immunoblot detection of an ~34 kDa outer membrane protein candidate from *Aeromonas* strain AHS. (A) Coomassie-stained 12% SDS–PAGE shows a dominant band at ~34–35 kDa (boxed). (B) Western blot of the same preparation displaying a major immunoreactive band at ~34 kDa (boxed). Molecular weight markers (kDa) at 30, 35, and 40 kDa are indicated on the left; M, marker; S, sample. Methods (Laemmli gel; Towbin transfer to nitrocellulose) were performed following the standard OMP workflows used in Aeromonas studies. Lanes are annotated with ladder positions, and the target band is boxed at ~34–35 kDa; for reproducibility, antibody reagents and dilutions are reported in the figure legend.

Coomassie-stained 12% SDS–PAGE of the *A. hydrophila* AHS preparation resolved a dominant band at ~34–35 kDa (Figure 2A) with annotated molecular weight ladder positions (kDa). Immunoblotting of the matched lane under standard Towbin transfer conditions revealed a single major immunoreactive species at ~34 kDa (Figure 2B) that co-migrated with the gel band. The concordant migration indicates that the abundant ~34 kDa species corresponds to the antibody-recognized antigen (hereafter referred to conservatively as native/putative Omp34 [nOmp34]). The gel–blot workflow and reporting are aligned with established OMP analyses in *Aeromonas* (Laemmli gel, Coomassie R-250; Towbin transfer to nitrocellulose), supporting methodological rigor. All vaccination lots used for in vivo studies satisfied the predefined QC thresholds (SDS–PAGE densitometry ≥80% purity; LAL <1 EU/μg; MBAS <0.01% w/v), as detailed in the Methods section. The ~30–50 kDa window is a typical size range for immunogenic *Aeromonas* OMPs, several of which have been validated as vaccine antigens (Omp34, Omp48), reinforcing the rationale for immunogenicity testing in this study; orthogonal peptide-level confirmation (LC–MS/MS) is pre-specified for follow-up without altering the present scope.

### Humoral immune response: Anti-Omp34 IgG2a kinetics

**Figure 3:**
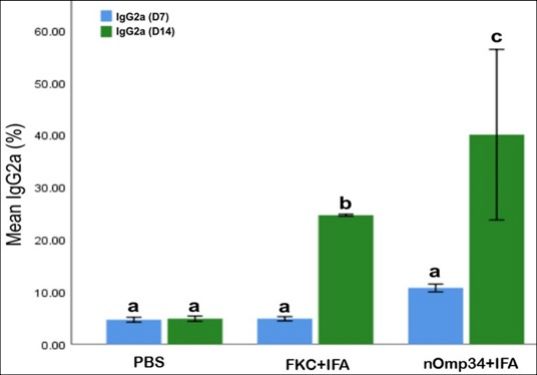
Anti-Omp34 IgG2a kinetics on days 7 (D7) and 14 (D14) post-immunization. Bars show mean ± SEM; blue = D7, green = D14. Tukey’s Honestly Significant Difference from one-way analysis of variance (α = 0.05): D7 all “a” (no differences); D14 nOmp34 + IFA “c” > FKC + IFA “b” > PBS “a” (all p < 0.05). SEM = Standard error of mean, FKC = Formalin-killed cells (vaccine), IFA = Incomplete Freund’s adjuvant, PBS = Phosphate-buffered saline.

On day 7, IgG2a levels were consistently low across all groups (Figure 3) with no significant differences between them (all marked “a”). By day 14, a distinct group effect became apparent: the nOmp34+IFA group achieved the highest average IgG2a level (~40%), surpassing FKC + IFA (~25%) and PBS (~5%). All pairwise comparisons were statistically significant (Tukey letters c, b, a; one-way ANOVA per day, p < 0.05). From day 7 to 14, increases within groups were notable for nOmp34 + IFA and FKC + IFA, but minimal for PBS; the wider variation in nOmp34 + IFA reflects individual differences rather than a loss of signal. The IgG2a dominance observed on day 14 suggests a Th1-biased humoral response aligned with antibacterial immune protection and supports the study’s aim to validate Omp–based vaccine candidates.

### Innate Immunity marker: Serum lysozyme dynamics

**Figure 4:**
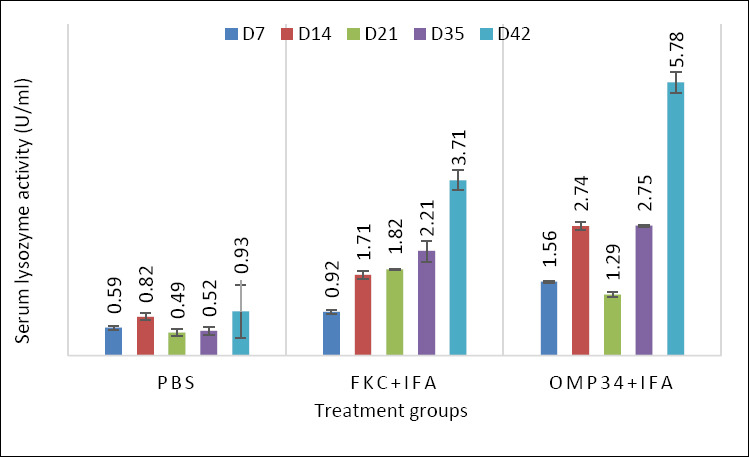
Kinetics of serum lysozyme activity after immunization. Serum lysozyme activity (U/mL) on days (D) 7, 14, 21, 35, and 42 following immunizations with nOmp34 + IFA, FKC + IFA, or PBS. Bars represent the mean ± SD (n = 3 per group/time). Statistical analysis was performed daily using one-way analysis of variance followed by Tukey’s Honestly Significant Difference (α = 0.05); different letters above the bars indicate significant differences (same letter = not significant). Observed patterns: D7 (nOmp34 + IFA > FKC + IFA > PBS), D14 (no differences), D21 (FKC + IFA > nOmp34 + IFA > PBS), D35–D42 (nOmp34 + IFA > FKC + IFA > PBS). D21/D35/D42 = day 21/day 35/day 42 (hari ke-21/35/42), FKC = Formalin-killed cells (vaccine), IFA = Incomplete Freund’s adjuvant, nOmp34 = native/putative outer membrane protein 34 kDa subunit, PBS = phosphate-buffered saline.

Serum lysozyme activity (Figure 4) showed a clear vaccine- and time-dependent pattern over 7–42 days (n = 3/group). One-way ANOVA within each day indicated significant group effects at D7, D21, D35, and D42 (F = 130–694; p ≤ 1.1×10^−5^; η² ≈ 0.98–1.00), but no difference at D14 (p = 0.178). Post hoc Tukey tests clarified the dynamics: at D7, nOmp34 + IFA > FKC + IFA > PBS (all p ≤ 0.0001); by D21, the order reversed to FKC + IFA > nOmp34+IFA > PBS (all p < 10^−4^). After boosting, nOmp34+IFA regained dominance at D35 and remained higher at D42, with 5.78 ± 0.16 U/mL versus 3.71 ± 0.22 (FKC + IFA) and 0.94 ± 0.58 (PBS). The differences between nOmp34 + IFA and FKC + IFA at D35 (p = 0.0079) and D42 (p = 0.0011), as well as all vaccine versus PBS comparisons (≤ 0.0002), were significant. This late, amplified increase, consistent with the cohort’s Th1-skewed IgG2a response, suggests durable priming of granulocyte/monocyte bacteriolytic function by the OMP subunit. Therefore, lysozyme levels at D35–D42 should be designated as a secondary endpoint and combined with NBT, phagocytic indices, and post-challenge survival/bacterial load within a multivariate framework to help establish an antigen-linked correlate of protection for the nOmp34 candidate.

### Innate immunity marker: Respiratory burst (NBT) activity

**Figure 5:**
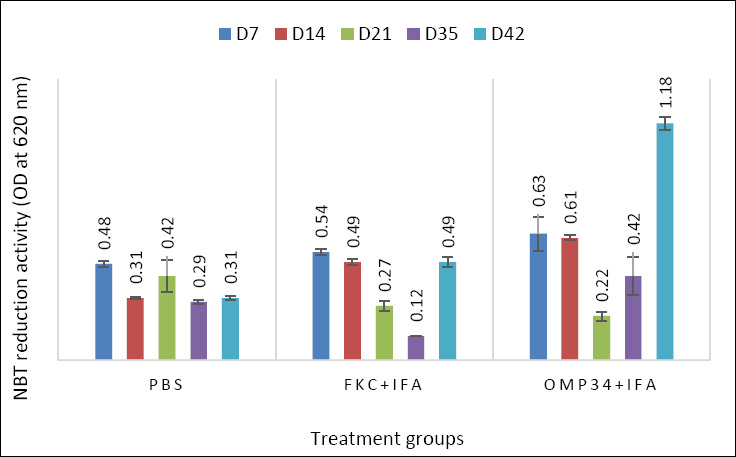
Respiratory burst activity measured by NBT reduction (OD_620_) in the experimental groups on days 7, 14, 21, 35, and 42 after immunization. Bars represent the mean ± SD (n = 3 per group/day). A one-way analysis of variance was conducted for each day, followed by Tukey’s Honestly Significant Difference (α = 0.05); different letters above the bars indicate significant differences (same letter = not significant). Across all time points, both vaccine groups surpassed PBS, and nOmp34+IFA consistently outperformed FKC + IFA, with the greatest separation observed on day 42.NBT = Nitroblue tetrazolium, FKC = Formalin-killed cells (vaccine), IFA = Incomplete Freund’s adjuvant, nOmp34 = native/putative outer membrane protein 34 kDa subunit, PBS = phosphate-buffered saline.

NBT reduction (Figure 5) respiratory burst activity (OD620) showed a vaccine- and time-dependent pattern: one-way ANOVA for each day found no difference at D7 (F = 4.99, p = 0.053), but strong group effects at D14 (F = 1133.3, p = 1.84×10^−8^, η² = 0.997), D21 (F = 14.02, p = 0.0055, η² = 0.824), D35 (F = 12.49, p = 0.0073, η² = 0.806), and D42 (F = 1529.5, p = 7.50×10^−9^, η² = 0.998). Tukey’s HSD identified clear differences with smooth phase shifts: at D14, the pattern was nOmp34+IFA > FKC + IFA > PBS (all p < 0.001), reflecting an early vaccine–induced oxidative burst; at D21, the order reversed to PBS > FKC + IFA = nOmp34+IFA (PBS vs FKC p = 0.0186; PBS vs nOmp34 p = 0.0056), indicating a temporary contraction after priming; by D35, only nOmp34+IFA > FKC + IFA remained significant (*p* = 0.0059), and at D42, the response consolidated as nOmp34+IFA > FKC + IFA > PBS, with all pairwise differences significant (p ≤ 0.0001), peaking in the nOmp34 group (1.180 ± 0.026 OD_620_). The letters above bars indicate Tukey groupings within each day (same letter = no significant difference). The late dominance of nOmp34 suggests sustained activation of NADPH-oxidase–mediated killing in neutrophils and monocytes, offering a mechanistically consistent innate signature for the OMP subunit. The D35–D42 NBT results should be combined with IgG2a/lysozyme levels and post-challenge survival data in a multivariate analysis to help identify an antigen-linked correlate of protection for this candidate.

### Innate effector function: Phagocytic capacity

**Figure 6:**
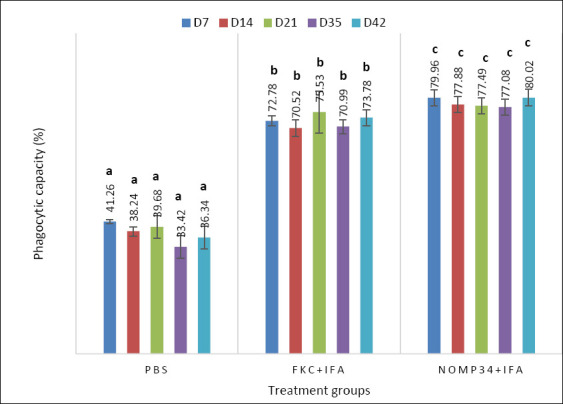
Phagocytic capacity (%) at days 7, 14, 21, 35, and 42 post-immunization in BALB/c mice treated with PBS, FKC, or nOmp34, each formulated in Freund’s adjuvant (CFA prime, IFA boosts). Bars indicate the mean ± SD (n = 3 per group per time point). One-way analysis of variance was conducted within each day, followed by Tukey’s Honestly Significant Difference (α = 0.05). Different letters above bars denote significant differences within the same day; bars sharing a letter are not significantly different.

Phagocytic capacity showed a consistent vaccine-driven enhancement from day 7 to day 42 (Figure 6). From D7 to D42, phagocytosis showed a consistent vaccine effect: one-way ANOVA with Tukey’s HSD at each time point revealed significant group differences, with a clear order of nOmp34+IFA (c) > FKC + IFA (b) > PBS (a). The nOmp34+IFA group maintained a high level of approximately 70–80% phagocytosis, FKC + IFA stayed intermediate at about 60%–70%, and PBS remained low at roughly 35%–45%. Narrow error bars indicate small within-group variation, highlighting the strength of these differences. Coupled with higher NBT and lysozyme readings, this pattern suggests sustained priming of opsonophagocytic function, consistent with increased neutrophil/ monocyte activity driven by OMP-specific immunity. It also supports considering D35–D42 phagocytic capacity as a secondary endpoint to be analyzed alongside NBT, lysozyme, and post-challenge results when establishing an antigen-linked correlate of protection for the nOmp34 candidate.

### Association between immune biomarkers and protection

**Figure 7:**
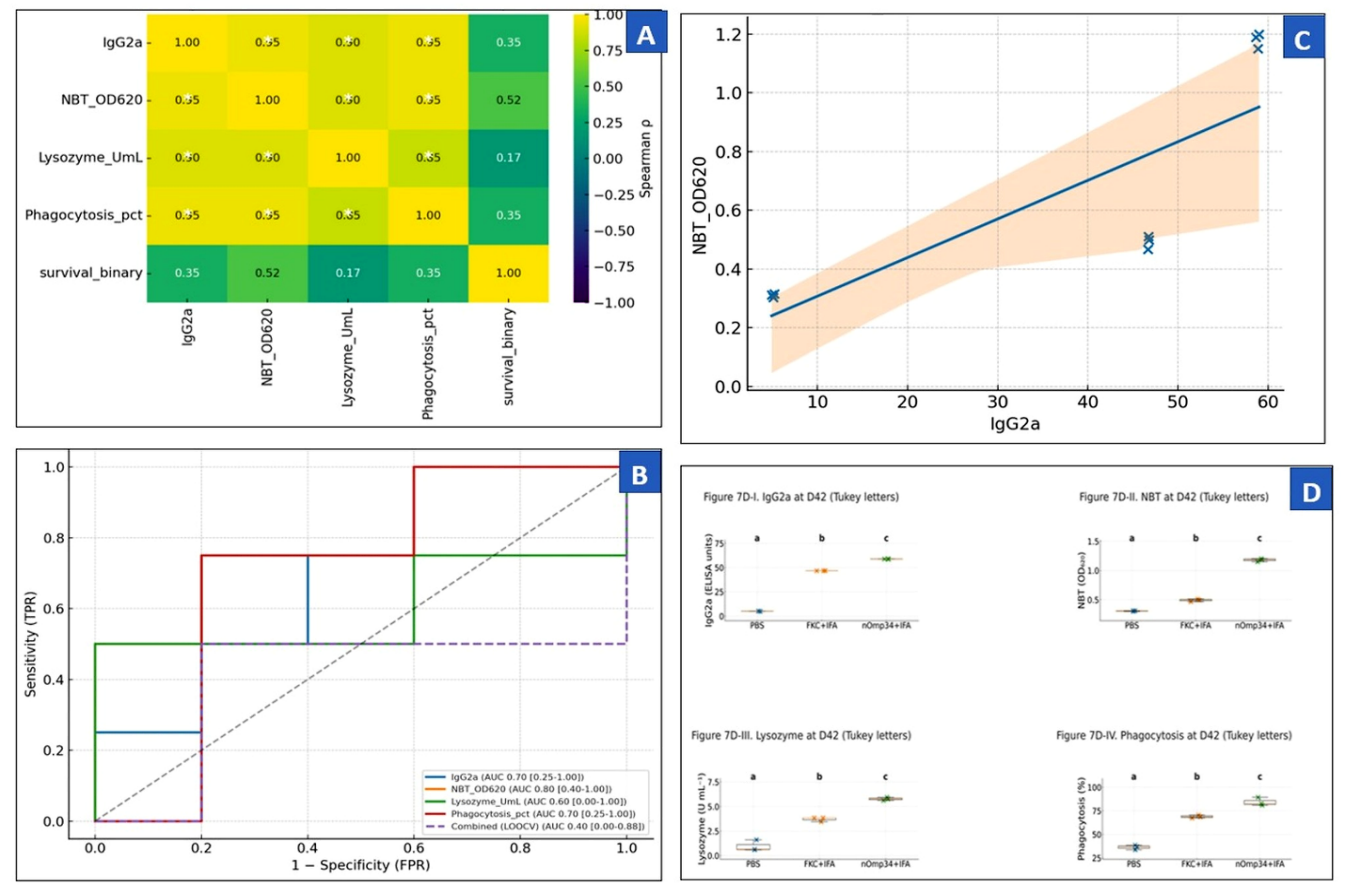
Integrative analysis of immune responses at day 42. (A) Spearman correlation matrix among IgG2a (ELISA units), NBT (OD620), lysozyme (U mL−1), phagocytosis (%), and survival (binary); cells display ρ, with * indicating p < 0.05. (B) ROC curves for each biomarker predicting survival; the legend reports AUCs with 95% CIs, and the dashed purple line shows the leave-one-out cross-validated combined model. (B′) ROC analysis of NBT for differentiating treatment groups versus PBS (nOmp34+IFA vs. PBS; FKC + IFA vs. PBS). (C) Scatter plot of IgG2a versus NBT colored by group with least-squares fits and 95% confidence interval; annotations show the overall Spearman ρ and the partial ρ controlling for group (ρ|group). (D) Boxplots with overlaid points for IgG2a, NBT, lysozyme, and phagocytosis at day 42; horizontal lines indicate medians, and letters denote Tukey’s Honestly Significant Difference groupings from one-way analysis of variance (α = 0.05).

At D42 (pre-challenge), all biomarkers distinctly differentiated treatment groups in the sequence nOmp34+IFA > FKC + IFA > PBS (one-way ANOVA with Tukey’s HSD, α = 0.05), with means (±SD): IgG2a, PBS 5.07 ± 0.14, FKC + IFA 46.71 ± 0.06, nOmp34+IFA 58.83 ± 0.17; NBT (OD_620_), 0.31 ± 0.01, 0.49 ± 0.02, 1.18 ± 0.03; lysozyme (U mL^−1^), 0.95 ± 0.56, 3.71 ± 0.21, 5.78 ± 0.16; phagocytosis (%), 36.34 ± 2.42, 68.85 ± 1.61, 84.35 ± 4.24 (Figure 7D; n = 3/group). Correlation mapping (Figure 7A) showed strong co-variation among biomarkers (Spearman ρ ≈ 0.85–0.95; p < 0.05) and positive correlation with day 14 survival (ρ ≈ 0.35–0.52), indicating a coherent innate–humoral response. Treatment-specific scatterplots (Figure 7C) demonstrated a graded IgG2a–NBT dose–response (ρ ≈ 0.95, p < 0.001) and, after adjusting for treatment, a smaller but consistent partial association (partial ρ|group ≈ 0.20), supporting the signal while tempering causal claims. Predictive analyses (Figure 7B) ranked NBT as the most informative single marker (AUC ≈ 0.80; 95% CIs via bootstrap on LOOCV probabilities), followed by IgG2a and phagocytosis (AUC ≈ 0.70), while lysozyme showed less discrimination (AUC ≈ 0.60); a simple combined score did not surpass NBT. A penalized multivariable logistic model including all biomarkers and treatment status (Table 2) retained a positive coefficient for NBT after adjustment and was consistent with an independent protective effect of nOmp34+IFA versus PBS. LOOCV AUC indicated acceptable discrimination. The model estimates (β, OR [95% CI], p, and AUC) are provided in Table 2. To operationalize, Youden-optimized cut-offs with sensitivity and specificity are included for each biomarker (Table 3); optional pairwise ROC comparisons between vaccine groups (nOmp34 vs. PBS; FKC vs. PBS) are summarized in Figure 7B′/Table S1a. Overall (Figures 7A–D; Table 2; Table 3), the D42 profile mirrors the post-challenge efficacy ranking (PBS < FKC + IFA < nOmp34+IFA) and supports the nOmp34 subunit as the most plausible candidate linked to protection in this dataset, with cautious conclusions given the modest sample size.

**Table 2:** Penalized multivariate logistic regression at D42 (all biomarkers + treatment). Reported: penalized sβ, OR (95% CI), and p-value from unpenalized logistic regression. PBS is marked as the reference level (no p-value by definition).

Predictor	Beta (penalized)	OR	95% Confidence interval	p-value
IgG2a	0.06	1.06	0.702–1.601	0.208
NBT_OD_620_	0.342	1.41	0.837–2.051	0.218
Lysozyme_UmL	–0.111	0.9	0.592–1.405	0.231
Phagocytosis_pct	0.028	1.03	0.638–1.648	0.247
PBS	0.003	1.0	0.559–1.791	(reference)
nOmp34+IFA	0.234	1.26	0.636–2.400	0.489

CI = Confidence interval

**Table 3:** Youden-optimized cut-offs per biomarker at D42 with sensitivity, specificity, and area under the ROC curve (95% CI).

Biomarker	Cut-off	Sensitivity (%)	Specificity (%)	Youden J	AUROC (95% CI)
IgG2a	5.19	100.0	40.0	40.0	0.700 (0.250–1.000)
NBT_OD_620_	0.511	75.0	80.0	55.0	0.800 (0.396–1.000)
Lysozyme_UmL	5.78	50.0	100.0	50.0	0.600 (0.000–1.000)
Phagocytosis_pct	70.6	75.0	80.0	55.0	0.700 (0.250–1.000)

CI = Confidence interval

### Protective efficacy following lethal challenge

**Figure 8:**
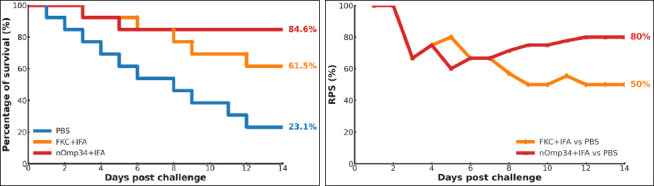
Post-challenge survival and vaccine protection (RPS). (A) Kaplan–Meier survival curves over 14 days post-challenge for PBS, FKC + IFA, and nOmp34 + IFA. Terminal survival was 23.1 % (PBS), 61.5 % (FKC + IFA), and 84.6 % (nOmp34+IFA). (B) Relative percent survival versus PBS plotted by day; higher values indicate greater protection. End-of-study RPS: 50 % for FKC + IFA and 80 % for nOmp34+IFA. Statistical comparisons (log-rank for panel A) were performed as described in the Methods section.

Following the LD_80_ *A. hydrophila* challenge (24–48 h after D42), Kaplan–Meier curves quickly separated and remained ordered: PBS < FKC + IFA < nOmp34+IFA (Figure 8A). By day 14, survival rates were 23.1% (3/13) for PBS, 61.5% (8/13) for FKC + IFA, and 84.6% (11/13) for nOmp34+IFA. Log-rank tests confirmed significant protection compared to PBS for FKC + IFA (χ² = 5.20, p = 0.0226; Cox HR = 0.31, 95% CI 0.10–0.91) and for nOmp34+IFA (χ² = 9.29, p = 0.0023; HR = 0.13, 95% CI 0.03–0.61). The direct comparison between nOmp34+IFA and FKC + IFA was not significant (χ² = 1.40, p = 0.238; HR = 0.39, 95% CI 0.08–2.00). Similarly, time-resolved RPS relative to PBS converged by day 14 to 50% for FKC + IFA and 80% for nOmp34+IFA (Figure 8B), highlighting the superior protective efficacy of the nOmp34 subunit over the whole-cell FKC benchmark. No grade ≥2 injection-site reactions were observed; however, occasional grade 1 erythema or ≤2 mm swelling resolved without intervention during days 1–7. Body weight remained within ±10% of baseline, and no significant group × time interaction was detected. Necropsy showed no consistent gross lesions at injection sites or internal organs, and histopathology was not performed.

### Safety and tolerability assessment

**Figure 9:**
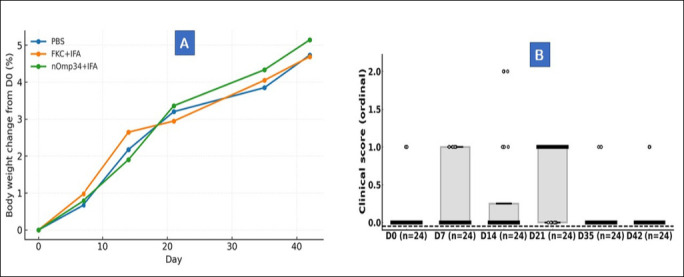
Safety and tolerability during the immunization phase (D0–D42). (A) Change in body weight from baseline (%), mean ± SEM: PBS (blue ●), FKC + IFA (orange ◆), nOmp34+IFA (green ▲); mixed-model p > 0.05 (no group×time effect). Dashed line: −10 % threshold. (B) Clinical scores (0–3) by day/group; boxplots with jittered points; Kruskal–Wallis p > 0.05 across days. Scores mostly 0–1; no humane-endpoint events; survivors’ necropsy unremarkable. n = 13/group.

Body weight trajectories remained stable and similar across groups throughout the immunization period (Figure 9A). No mouse experienced ≥10% weight loss from baseline, and group averages gradually increased by approximately 2%–5% by day 42. Clinical scores stayed low (median = 0, IQR = 0–0) with only occasional grade 1 events and a single transient grade-2 observation; no humane endpoints were reached (Figure 9B). Differences between groups in body weight change and clinical scores were not statistically significant (p > 0.05), indicating good local and systemic tolerability of both FKC + IFA and nOmp34 + IFA.

## DISCUSSION

### Study objectives and taxonomic context

This study aimed to determine whether a specific ~34 kDa OMP (nOmp34) from an Aeromonas isolate, provisionally classified within the *A. hydrophila* group, (i) induces a consistent day 42 (D42) innate–humoral response and (ii) converts that signal into post-challenge protection while remaining well-tolerated. Our 16S rRNA neighbor-joining (K2P) analysis placed the AHS isolate within the *A. hydrophila* sensu lato cluster with typical support for shallow Aeromonas 16S separations; due to the limited intraspecies resolution of 16S, we regard this placement as provisional and will verify species-level identity through MLSA (gyrB, rpoD, recA) or, ideally, WGS/ANI [28–30].

### Characterization of the putative Omp34 antigen

Our 12% SDS–PAGE followed by Towbin immunoblot consistently revealed a single, dominant immunoreactive species at 34–35 kDa, squarely within the canonical OMP window. Mechanistically, OMPs are β-barrel, surface-exposed antigens whose solvent-accessible loops promote B-cell recognition and major histocompatibility complex presentation. This property helps explain why OMP subunits (tOmpN/OmpW/ OmpA/Omp38/Omp46/Omp48) often outperform bacterins [16, 21–25, 31, 32]. Consistent with this paradigm, recent immunoproteomic studies have repeatedly identified OmpA/38/46/48-like maltoporin proteins in *A. hydrophila* as immunogenic and protective, reinforcing the biological plausibility of our signal. Taken together, the clear 34–35 kDa band is consistent with an authentic Omp34-like immunogenic protein; therefore, we will confirm its identity using LC–MS/MS before cross-strain epitope mapping and sequence-guided refinement, employing reverse vaccinology and multi-epitope design to enhance coverage, adjuvanticity, and manufacturability [16, 21, 32]. This stepwise process transforms a strong biochemical observation into a well-defined, traceable OMP subunit candidate.

### Day 42 immune signature (humoral and innate)

The D42 immune profile clearly distinguished between groups (nOmp34+IFA > FKC + IFA > PBS) across IgG2a, NBT, lysozyme, and phagocytosis. This reflects a tightly coordinated innate–humoral response (Spearman ρ up to 0.95) and a Th1-leaning pattern in BALB/c mice, where IgG2a is a key indicator [33, 34]. These measures are standard in aquatic vaccinology: NBT assesses the NADPH-oxidase respiratory burst, turbidimetric lysozyme (with *M. lysodeikticus*) measures bacteriolysis, and microscopic phagocytosis evaluates cellular uptake, all indicating innate immune priming before challenge [35–39]. Consequently, time-to-event efficacy supports immunogenicity: Kaplan–Meier survival and RPS rank PBS < FKC + IFA < nOmp34+IFA, a pattern and metric commonly used in finfish vaccine studies [40, 41], where survival metrics and RPS are standard [42, 43]. The better performance of nOmp34+IFA matches evidence that OMP antigens can promote protective antibacterial immunity in teleosts [40, 44].

### Translational relevance across host systems

Although all efficacy and biomarker analyses were conducted in BALB/c mice, the integrated pattern, including respiratory burst (NBT superoxide (O_2_•−)), lysozyme, phagocytosis, and IgG2a, maps onto conserved antimicrobial modules expected in teleosts. Therefore, we treated the mammalian model as hypothesis-generating to focus on practical fish correlates (field-deployable respiratory burst and phagocytic indices) that, once validated, can support antibiotic-sparing vaccination aligned with WHO AWaRe stewardship in aquaculture [45–47]. Within this framework, NBT served as the primary correlate of survival in this dataset. To facilitate translation, we will perform a pre-specified teleost program using standardized NBT superoxide (O_2_•−) and phagocytosis assays, bacteriological endpoints (organ CFU and reisolation), and multivariable modeling with external replication where feasible [48, 49]. Simultaneously, safety monitoring (blinded 0–3 injection-site scoring, body weight, and clinical scores) showed no grade ≥2 local reactions, stable weight (±10%), and low clinical scores; although serum biochemistry was not collected, a predefined systemic panel (ALT, AST, BUN/creatinine, CK, ± basic hematology) at fixed post-dose and post-challenge windows will be added to complete tolerability profiling [47, 48].

### Comparative performance against the formalin-killed bacterin

In our within-study benchmark, the native/putative Omp34 (nOmp34) subunit outperformed the formalin-killed bacterin (FKC) under matched conditions (host, adjuvant, route, LD_80_-calibrated inoculum) [15]. Although recombinant OMP vaccines (e.g., OmpA/OmpW/Omp48) in fish models have shown a range of RPS values across species, adjuvants, doses, and challenge designs [15, 22, 31, 44], directly comparing these to our murine results is not methodologically appropriate [4]. Nonetheless, the magnitude and consistency of protection seen in this study align with those of defined-antigen candidates tested under controlled protocols [15, 22, 31]. To improve translational relevance, our future work will include a direct comparison with a recombinant OMP using matched adjuvant and antigen mass, along with a fish-relevant delivery platform (e.g., PLGA, outer membrane vesicles, or a feed-compatible formulation) [50, 51], with efficacy assessments extending beyond survival to include bacteriological endpoints [52].

### Biomarker–outcome associations and mechanistic interpretation

The D42 association analyses linked immune responses to outcomes: ROC identified NBT as the most informative single marker with an AUC of about 0.8, and penalized logistic regression kept NBT after adjustments, supporting an oxidative-killing mechanism [53]. This aligns with evidence that neutrophil respiratory bursts and downstream NETs are key to early containment of invasive bacteria [54, 55]. In Aeromonas, sodA/sodB–mediated detoxification of phagocyte-produced ROS allows bacteria to persist within phagocytes, explaining why a strong NBT signal would correlate with survival [56]. Additionally, IgG2a-mediated opsonophagocytosis likely adds to this effect through FcγRIV engagement on neutrophils and monocytes [57, 58], which matches foundational work showing the IgG2a/2b–FcγRIV pathway as a powerful effector mechanism [59].

### Safety, tolerability, and preclinical compliance

The safety and tolerability were acceptable and aligned with ARRIVE 2.0 expectations for well-tolerated preclinical vaccine studies [60]. Translationally, the numerical advantage of nOmp34+IFA over FKC + IFA supports recent findings that defined OMP antigens, when stabilized in suitable delivery systems, can match or surpass inactivated bacterins. This validation encourages progressing nOmp34 into more deployable platforms (such as OMV/vesicles, liposomal/LEPS, or other non-Freund’s adjuvants) and expanding coverage across strain heterogeneity on farms, which could help reduce antimicrobial use [40, 61].

### One Health and cross-species vaccine implications

Within a One Health framework, we tested a fish pathogen in a mammalian immune model and, to our knowledge, provided proof-of-concept for the translational potential of an Omp34 subunit vaccine, which may guide antibiotic-sparing strategies in aquaculture [62–64]. The Omp34 formulation showed efficacy in BALB/c mice; this represents an unusual direct comparison of a specific subunit versus a whole-cell vaccine in a mammalian model [22, 65–67]. Using a mammalian surrogate also allowed mechanistic studies, which are difficult in teleosts, especially the innate oxidative and opsonophagocytic pathways that underpin OMP-mediated protection [68–70]. Translationally, Omp34-like β-barrel antigens are expected to show cross-species loop-level conservation among Aeromonas isolates from fish and mammals, consistent with genomic and structural evidence for conserved OMP families across hosts [63]. The current native-antigen workflow can be scaled up using standard biochemistry steps, and recent reviews on aquaculture vaccines, which outline practical delivery routes and deployment considerations without changing the core antigen strategy, support field implementation [63, 64, 71].

### Methodological advances and analytical framework

Methodologically, we developed a native-antigen workflow that isolates β-barrel Omp34 directly from bacterial membranes using sarcosyl + DDM and detergent-assisted refolding, preserving conformational epitopes and minimizing endotoxin carry-over in a simple, scalable manner, unlike typical recombinant pipelines [72–74]. We utilized an integrated inferential framework to identify independent immune correlates of survival, ensuring robustness beyond univariable associations [75–77].

### Future directions and knowledge gaps

The data support heterologous challenges with various Aeromonas isolates, epitope mapping of conserved β-barrel loops for multi-epitope design, and a staged translational program that implements these platforms within coordinated study designs [16, 60, 78, 79]. Simultaneously, we will implement field validation of NBT and lysozyme as surrogate biomarkers to speed up vaccine trials [67, 80, 81]. We note that directly evaluating target fish species and antibiotic use outcomes is beyond the current scope; therefore, the claims remain exploratory. However, the results indicate a feasible translational link between rodents and aquaculture immunology [82, 83].

### Limitations and final interpretation

Our proof-of-concept study has four main limitations: limited statistical power for immunological endpoints, provisional taxonomy (16S rRNA only), restricted adjuvant generalizability (IFA), and absence of post-challenge organ-burden readouts. Direct inferences about pathogen clearance are therefore limited. A preregistered follow-up will include organ CFU/g with reisolation, blinded histopathology, expanded isotyping, and functional humoral assays (OPK/SBA). We will also verify species-level taxonomy using WGS/ANI or MLSA, confirm the antigen identity of the ~34 kDa band with LC–MS/MS, and assess deployable formulations in larger, species-relevant studies with heterologous challenges and dose–response, employing D35–D42 NBT plus RPS as co-primary endpoints; conformation (CD/native-PAGE), β-barrel topology, and freeze–thaw/−80°C stability will be verified against predefined criteria. Even with these limitations, the day 42 signature (IgG2a↑, NBT↑, lysozyme↑, phagocytosis↑) offers a biologically relevant link to survival. Point estimates favored nOmp34 over FKC under matched conditions, but the direct comparison was not statistically significant; nonetheless, nOmp34 remains a prioritized, correlate-linked candidate for aquaculture scale-up and cross-protection testing.

## CONCLUSION

This study demonstrates that nOmp34 from an *Aeromonas* isolate, which is provisionally classified within the *A. hydrophila* group, triggers a coherent and mechanistically meaningful immune response in BALB/c mice. It also provides measurable protection against a near-lethal challenge. In all immunological assessments conducted on day 42, such as IgG2a levels, NBT respiratory burst, serum lysozyme, and phagocytic capacity, the nOmp34+IFA formulation consistently outperformed both the formalin-killed bacterin (FKC + IFA) and PBS control. This indicates a tightly coordinated innate–humoral response. The survival data support these findings: nOmp34+IFA achieved the highest protection rate at 84.6%, followed by FKC + IFA at 61.5%, both surpassing PBS at 23.1%. The RPS aligned accordingly at 80% and 50%. Further association analyses identified NBT respiratory burst as the strongest single predictor of survival, consistent with its role in oxidative-killing mechanisms critical for early antibacterial defense.

Practical implications include the feasibility of using innate biomarkers, especially NBT and phagocytic indices, as field-deployable indicators of protection for aquaculture vaccine screening. The results also support the translational relevance of mammalian models for mechanistic investigation of OMP–based vaccines, where teleost assays remain limited. The strong performance of nOmp34 aligns with a growing body of evidence showing that defined OMP antigens can match or outperform traditional bacterins and therefore serve as promising candidates for antibiotic-sparing immunoprophylaxis in aquaculture systems under WHO AWaRe stewardship principles.

Key strengths of the study include: (i) a native-antigen workflow that preserves conformational epitopes through sarcosyl extraction and DDM-assisted refolding; (ii) a rigorous blinded design with validated QC thresholds; (iii) the integration of multiple immunological endpoints; and (iv) the use of penalized multivariable modeling to identify correlates rather than relying solely on univariable associations. These elements provide a strong methodological foundation for advancing Omp34-like antigens in translational vaccine development.

Limitations include a provisional species-level classification based only on 16S rRNA, limited statistical power for some immunological endpoints, restricted generalizability due to the use of IFA as an adjuvant, and the lack of post-challenge organ-burden or histopathological confirmation of bacterial clearance. The study was also conducted solely in mice, so direct extrapolation to fish species should be approached cautiously.

Future research will focus on confirming isolate taxonomy using WGS/ANI or MLSA, validating the identity of the approximately 34 kDa antigen via LC–MS/MS, and expanding evaluation to recombinant OMP formats and fish-appropriate delivery systems such as PLGA microparticles, OMVs, or oral/feed-compatible formulations. Heterologous challenges, conserved epitope mapping, dose–response assessment, and bacteriological endpoints (organ CFU and reisolation) will be incorporated into a harmonized translational pipeline. Additionally, NBT and lysozyme assays will be operationalized as surrogate biomarkers to speed up screening in aquaculture settings.

In conclusion, the native/putative Omp34 (nOmp34) subunit demonstrated strong immunogenicity, effective innate–humoral interactions, and superior protective efficacy under controlled conditions, outperforming the benchmark bacterin. Although exploratory, the findings suggest that nOmp34 is a promising, correlate-linked vaccine candidate with potential for reducing antimicrobial use in aquaculture. The results provide a biologically-based framework for initiating larger, species-specific validation studies, supporting the broader One Health objectives of pathogen control and antimicrobial stewardship in aquatic food production systems.

## DATA AVAILABILITY

All the generated data are included in the manuscript.

## AUTHORS’ CONTRIBUTIONS

RZ and SW: Conceived and designed the study. WT, JR, EBAH, MY, MAA, and SK: Developed the methodology and conducted the investigations. JR, MAA, and SK: Curated the data. RZ, MY, and AS: Performed the formal analysis, and RZ and AS: Created the visualizations. RZ and JR: Drafted the manuscript. WT, EBAH, and SW: Critically reviewed and edited the manuscript. WT and SW: Validated the findings. SW and WT: Supervised the study and drafted the manuscript. All authors have read and approved the final version of the manuscript.
